# Quality of sexual and reproductive health services for adolescents and young people in public health facilities in Southwest Nigeria: a mystery client study

**DOI:** 10.1080/16549716.2022.2145690

**Published:** 2022-12-02

**Authors:** Olujide Arije, Jason Madan, Tintswalo Hlungwani

**Affiliations:** a Institute of Public Health, Obafemi Awolowo University, Ile-Ife, Nigeria; b School of Public Health, University of Witwatersrand, Johannesburg, South Africa; c Warwick Medical School, University of Warwick, Warwick, UK

**Keywords:** Maria Emmelin, Quality of care, youth-friendly services, adolescent sexual and reproductive health, mystery client research, sentiment analysis

## Abstract

**Background:**

To support the policy drive for the promotion of sexual and reproductive health (SRH) of adolescents and young people (AYP), it is necessary to understand the characteristics of the existing SRH services available to them.

**Objective:**

To assess the provision and experiences of care in SRH services for AYP in a Nigerian setting.

**Methods:**

Twelve male and female mystery clients (MCs) conducted 144 visits at 27 selected primary and secondary health facilities in two Local Government Areas (LGA) in Ogun State, Nigeria. A 27-item adolescent quality of care (AHQOC) index with a Cronbach’s Alpha of 0.7 was used to obtain a quality-of-care score for each clinic visit. Linear panel-data random-effects regression models using the generalised least square estimator were used to assess quality associated factors. Sentiment analysis was done on the qualitative narrative summaries provided by MCs after each visit.

**Results:**

There was an absence of the use of educational materials during the 60.4% of the visits. The MCs’ medical history (90.3%), social record (63.9%), sexual/reproductive history (53.5%), and contraceptive experience (66.0%) were not obtained in most of the visits. Female MC visits had a lower AHQOC index rating on average compared to males (β=-0.3, CI −1.6 – 1.0 p = 0.687), rural health facilities had a lower AHQOC index rating on average compared to urban (β=-2.7, CI −5.1 – −0.2, p = 0.031), and a higher ranking of the health worker on the scale of 1–10 corresponded to a higher AHQOC index of the MC visit (β = 1.9, CI 1.6–2.1, p < 0.001). There were more positive than negative sentiments about the clinic encounters.

**Conclusion:**

This study found gaps in the competencies of the health workers, non-usage of educational materials in clinic encounters with young people, as well as the differential perception of quality of care by male and female AYP.

## Background

The World Health Organization (WHO) defines the quality of care as the extent to which health care services provided to individuals and patient populations improve desired health outcomes [1]. Quality of care can also be seen as the degree, grade of excellence, or worth of all inputs, processes, output, outcomes, and the impact of health service delivery [2]. The WHO quality of care framework for maternal and newborn health [3], a service-specific framework, helps in exploring adolescents’ health services because of its robustness. The framework is based on a structure-process-outcome model with key domains that fall under the provision of care and the experience of care, in addition to the provision of human and material resources [4]. The domains of the framework align with how adolescents and young people (AYP) are said to perceive health care, including accessibility, attitude of staff, communication, medical competency, age-appropriate environment, and health care involvement [5]. Provision of care is concerned with the technical components of care including evidence-based practices for routine treatment, management of complications, actionable information systems, and functional referral systems. In contrast, the experience of care relates to effective communication, respect and dignity, and emotional support provided while accessing health services [3]. Experience of care is also the subjective perception about health services, that is, the patient/user’s perspective and expectations, as well as individual health outcomes [6].

In context, the policy environment for AYP’s health represents the overarching health system within which care is provided. Current health policies and strategic frameworks have far-reaching consequences for providing acceptable care quality. In addition, service characteristics, care experience, and individual characteristics of adolescents (such as knowledge about services, family settings, and finances) likely influence user preferences for healthcare. Similarly, users’ preferences probably drive service utilisation, and ultimately user outcomes, which loops back to influence the experience of care of users. The combined observations of consultations, an indication of provision of care, and client exit interviews reflecting care experience, can therefore adequately represent the quality of care assessment [7].

Some authors have argued for using standardised patients (also called mystery clients) as the gold standard for quality of care measurement in health settings [8]. The mystery client (MC) method uses a trained observer to record their experience of service provision [9]. In the approach, the observer poses as a regular user of the service of interest without the service provider being aware. The method is reported to be reliable, valid, feasible, and acceptable [10,11]. This is because it reduces client courtesy/social desirability bias, behaviour modification under observation (Hawthorne effect), and poor recall among clients. Tumlinson et al. [12] demonstrated consistent overestimation of quality when using other methods such as provider interviews, client interviews, and observation of client-provider interactions to assess the quality of family planning services. The mystery client (MC) approach has been used in adolescent health research in different contexts including investigating family planning services [13], sexually transmitted infection (STI) treatment services, and HIV services [14]. For example, Mchome et al. [10] adopted the MC approach to evaluate the youth friendliness of reproductive health services in Tanzania as part of a baseline situation analysis to implement youth-friendly reproductive health programmes.

Ogun State, Southwest Nigeria, is at a policy junction with the introduction of the *Ogun State Adolescents and young people’s sexual and reproductive health strategic framework, 2018 – 2022* in 2019 [15]. The goal of Ogun State is that by 2022, AYP in the State should have access to correct information and services related to sexual and reproductive health (SRH). This policy direction presents an opportunity for grounding its implementation in research that explores the experiences of AYP concerning SRH services. To support the policy direction, it is necessary to understand the characteristics of the existing SRH services for adolescents in the State. Such understanding may facilitate the uptake of corrective actions that will improve the policy and service environment of AYP SRH. The objective of this research was thus to assess the provision and experiences of care in sexual and reproductive health services for AYP in public health facilities, and its associated factors in Ogun State. We used MCs to assess this provision and experience of care as a proxy for determining the quality of sexual and reproductive health services for adolescents. This study is a part of a more extensive study assessing the demand and supply sides of sexual and reproductive health services for AYP in Southwest Nigeria.

## Methodology

### Study design and location

We conducted a descriptive cross-sectional study of the quality of care in selected primary and secondary health facilities. Ogun State is one of the six states in the Southwest geopolitical zone of Nigeria [16]. Ogun State had an estimated population of 3,728,098 at the time of the 2006 national population census [17]. With a 2.5% annual growth rate, the population of Ogun State is currently estimated at 6,090,740 [18]. The predominant language of the people of Ogun State is Yoruba, with the individual sub-ethnic groups of the State speaking different dialects [16,19]. Adolescents and young people make up 30.7% of the State’s population [17]. Ogun State has three senatorial districts that share 20 Local Government Areas (LGAs), and this study was carried out in a predominantly urban LGA (Abeokuta South LGA) and rural LGA (Ijebu East LGA).

### Study health facilities

In this study, we assessed selected public primary and secondary health facilities across two of the 20 LGAs in Ogun State. We purposively selected Abeokuta South LGA, a predominantly urban LGA, and Ijebu East LGA, a predominantly rural LGA. Of the 16 public primary health care (PHC) facilities and three public secondary facilities in Abeokuta South LGA we selected 13 and two respectively. An additional two PHC facilities were selected as replacement facilities for two MCs because of concerns about being recognised. Of the 23 public PHC facilities and two public secondary facilities in Ijebu East LGA, we selected 11 and one respectively. These gave a total of 27 health facilities (24 PHC, and three secondary facilities) that we assessed. We considered all the available facilities eligible but the assessed PHC facilities were selected by systematic sampling while the secondary facilities were selected purposively. Primary Health Care (PHC) facilities operate at the community level and are supposed to be the first point of contact for clients. The secondary health facilities provide intermediate healthcare in general hospitals, comprehensive health centers, and general hospitals. The functions of primary and secondary facilities overlap significantly in many places within the country because of resource constraints, with secondary facilities taking up some of the functions of PHC facilities.

### Mystery client recruitment and training

Twelve MCs (six males and six females) in the age group of 18–24 years were recruited and trained for this study. They were students of two Schools of Nursing within the State. We considered that their prior knowledge would make them objective observers of clinical interactions with healthcare providers. This is similar to the approach of Nalwadaa et al. [13] who used graduate midwives in their MC study. The recruited persons were trained for three days using a purpose-designed training manual. The training was for mastering scripts developed from selected scenarios that covered common sexual and reproductive needs of AYP (see Box 1). The scenario on requesting information regarding sexually transmitted infection (STI) was only presented by male MCs. Counseling regarding premarital intercourse was presented by female MCs while both condom requests and requesting information regarding contraceptives were presented by both male and female MCs. During the training, the scenarios were translated into scripts that captured the picture of the typical AYP in the study location. The scripts defined each case in detail to standardise them and ensure comparability across providers. The script also included a short biography that described the social background, age, occupation, family details, and individual circumstances in the scenarios presented.

**Table:** 

**Box 1. Mystery client scenarios.Requesting information regarding STI** An 18/19-year-old male wants information regarding Sexually Transmitted Infections (STIs). His motivation for coming to the clinic is that he heard of a guy who got an STI from having intercourse with a commercial sex worker. Sexual history: he is sexually active and has had intercourse with approximately five women. Contraceptive use: he has used condoms only occasionally and does not like them. Reproductive health knowledge: minimal, has heard of a few diseases. The only symptom he has heard of is itching. He has heard that AIDS can kill you and thinks that AIDS only affects prostitutes. **Condom request:** The youth (19/20 years old) went to the health unit for condoms because s/he is sexually active. S/he has heard about STIs/HIV and that condoms may prevent them. The youth also heard that they are accessible at health units. However, the youth has heard many strange rumors about condoms, including the notion that condoms contain HIV or that they have holes. **General Family Planning:** 16/17-year-old school-going youth visits the facility to obtain information on family planning services. The youth tells the provider that s/he has never had intercourse before. A schoolmate has been her/his girlfriend/boyfriend for six months. Currently, the youth is feeling pressured by her/his girl/boyfriend to have sexual intercourse. The youth believes that if she/he does not accept s/he will leave her/him. S/he wants to avoid pregnancy to herself/his partner but knows very little about contraceptives. The youth has only heard of the pill and has many erroneous ideas about its use and possible side effects. The youth does not dare to talk with her/his parents about the problem because they are very strict and would not appreciate this kind of relationship. **Young adult female feeling pressured to have intercourse:** A 16/17-year-old woman comes to the facility to obtain birth control. She tells the provider that she has never had intercourse before. It becomes apparent that she is unsure if she wants to have intercourse, but she feels pressured by her boyfriend to do so. She believes if she does not give in, he will leave her, but she does not feel ready to initiate sexual activity. Her parents are very strict; she does not want them to know anything about this. Her partner: a schoolmate, has been her boyfriend for one year. Sexual history: none Contraceptive knowledge: minimal, has only heard of the pill, and has many erroneous ideas about its use and possible side effects.^1^

^1^Adapted from Boyce C, Neale P. Using mystery clients: a guide to using mystery clients for evaluation input. Pathfinder International; 2006.The training of the MC included an introduction to the MC methodology, a review of MC scenarios for the study, a session for scripting and narrative development of cases for the MC scenarios, group enactment of scripted cases, an introduction to the exit questionnaire to be administered to each MC after each clinic encounter, and risk mitigation during clinic encounter. In addition, each MC was trained to assess the environment of the facilities, staff, and healthcare provider behaviour, effectiveness of communication, respect and preservation of dignity, and emotional support provided. MCs were trained not to give the healthcare provider additional information outside the scripts or give information that was not prompted to avoid improvisation that may depart from the script they are to enact. Subsequently, each MC was required to pilot the assigned MC case in a health facility not included in the ones selected for the research.

### Data collection

A work plan was created to assign health facilities and days of visit to each of the MCs. Each of the six MCs in each LGA visited all the selected health facilities in their study LGA. No two MCs visited the same health facility on the same day. This was to limit the chances of being detected as an MC by a health worker who may recognise a pattern of a particular type of client on any of the days of data collection. On each assigned day, the MCs visited their assigned health facility, enacted the script as assigned to them during the MC training, then responded to a structured encounter interview after completing the visit, before visiting the next assigned health facility. In each LGA, a supervisor was assigned to administer the encounter interview to the MC after each clinic encounter at an agreed place far away from the visited clinic. Routine group debriefing meetings were held virtually to assess work done and address any emerging issues such as the possibility of detection and other research logistics.

### Data collection instrument

#### Quantitative instrument

The MC debriefing questionnaire had two main sections. The first covered characteristics of the health facility visited (name, location, level of facility), and the mystery client (age, sex). The second section included a 27-item quality of care scale with five subscales. The question items on the *‘Accessibility’* subscale focused on ease of locating the health facilities while those on the *‘Acceptable services’* subscale focused on whether young people find health facilities’ environment, setting, and procedures appealing and acceptable. For the ‘*Competencies and motivation of health worker’* subscale, the focus was on whether service providers were skilled and motivated to provide health services to young people in an adolescent/youth-friendly manner. Concerning the ‘*Privacy and confidentiality’* subscale, questions were asked about whether service providers were sensitive to the needs of young people and maintained their privacy and confidentiality in service provision. These thematic areas were derived from the Nigerian *National Standard and Minimum Package for Adolescent Healthcare* [20]. An additional theme was a *‘Global assessment’* subscale that asked general questions about the MC visit, including one to rank the health worker’s proficiency on a scale of one to ten. The 27-item adolescent quality of care (AHQOC) index was derived from an initial 37-item tool following factor analysis, reliability testing, and intraclass correlation coefficient test, to test for consistency. The original question items were derived from the mystery client exit questionnaires of Kwan et al [8] and Boyce and Neale [21]. The first is a manual for all types of mystery client studies while the second is a manual specifically for adolescent and young people mystery client study. Some question items were also derived from the Adolescent Client Exit Interview tool in WHO Global Standard for quality healthcare service for adolescents [22]. The Cronbach’s Alpha of 27-item was 0.7. The construction and the validation of the index are reported elsewhere.

#### Qualitative instrument

To capture some qualitative context of the MC visits, each MC was required to complete an online MC visit summary tool which consisted of some unstructured survey questions. They were required to complete this survey within 12 hours of the visit to the respective health facility while the experience of the visit was still fresh in their memories and certainly before visiting the next facility on their schedule. The questions asked were open-ended questions about various themes of service acceptability, competency, and motivation of health workers, privacy and confidentiality, and a global assessment of their clinic visit. These themes are the same as those assessed by the debriefing questionnaire.

Both the quantitative and qualitative data were collected and managed using REDCap electronic data capture tools hosted at the University of Witwatersrand [23,24].

### Data analysis

The responses to the 27-item AHQOC index are presented in frequencies and proportions. The AHQOC score was derived as the sum of the scores of the 27 items for each MC clinic visit. This study data has a panel structure since each health facility was assessed multiple times. Simple and multiple linear panel-data regression models that fit random-effects models by using the generalised least square (GLS) estimator, and with robust estimation of variance, were used to assess factors associated with the AHQOC score. The equation below expresses the basic structure of the regression model [25].

$$Quality_{ijkt}=\alpha +\beta *Provider_{it}+\gamma *MC_{jt}+\delta *Facility_{kt}+\epsilon {\;}_{ijkt}$$

*Provider*_*i*_ is a vector of provider characteristics at time t. The only provider characteristic assessable by the MC was the provider’s sex. *MC*_*j*_ is a vector of the MC characteristics at time t, including sex, age, and MC scenario. *Facility*_*k*_ is a vector of health facility characteristics at time *t*, including facility location (rural or urban) and care level (primary or secondary). ϵ is an error term. For this analysis, the health facility was the panel data variable. We included an interaction term of sex of the MC and the health workers to further explore their relationship in both the simple and the multiple linear regression models. All data were analysed using Stata/SE 15. The R-squared for the multiple linear panel-data regression model was 0.64

The narrative summaries in the completed online open-ended questionnaire survey completed by MCs were downloaded into MS Excel files, specially formatted, and imported into ATLAS.ti 9 using the ‘Import survey data’ function. Thematic codes (acceptability of services, competence and motivation, privacy and confidentiality, and global assessment) were created and linked with the full responses to each thematic question. Subsequently, thematic sub-codes were created and linked to relevant subsections within each of the thematically coded data segments. Each of the coded subsections was also tagged as either a positive or negative sentiment. Coding was done by an external qualitative data analyst with up to five years of experience in coding transcripts in qualitative research; the coding was revised by OA. We conducted a sentiment analysis as a distribution of positive and negative sentiments for each thematic area.

### Ethical considerations

This study was approved by the Human Research Ethics Committee of the Ogun State Primary Health Care Development Board (OGHECADEB) Ethics Committee (#OGPHC/021/008) and the University of the Witwatersrand (#M210315). The mystery clients were recruited from the Schools of Nursing Ijebu-Ode and Abeokuta in Ogun State with the permission of the respective School’s Principal. Their participation was voluntary, and all were at least between 18–24 years old. They were all required to complete a written informed consent to participate. Each of the mystery clients received a standard field allowance for research assistants during the fieldwork. They were also provided with transportation and communication allowance. The Medical Officer of Health in each LGA was informed about the research about two weeks before the commencement of data collection. They were requested to broadcast the information about an intended mystery client survey within the LGA on the central communication platform for all facility heads in the LGA. However, the specific health facilities or times of visit were not disclosed. Risk mitigation training was also included in the MC training since the MCs were exposed to the risk of being requested for physical examination as part of their consultation with service providers. We trained the mystery clients to refuse the option of physical examinations should they occur at the clinic encounters.

## Results

### MC visit characteristics

One hundred and forty-four MC visits were completed by 12 MCs, with each conducting 12 visits. The original design was to have equal numbers of health facilities in the two study LGAs. However, some MCs had to have their scheduled health facilities replaced because of concerns about being recognised. The MCs reported no adverse events during the facility visits. All the MCs felts they presented their cases as scripted. The ages adopted for the MC scenarios ranged from 16–20 years of age and the sex of the MCs was balanced equally between males and females. However, 88.2% of the encounters the MCs had were with female health workers.

### Opinions about the health facilities

For most of the visits conducted, the MC found the facility easily, with directional signs outside the facility (Table 1). However, directional signs were not seen within the facilities in 55.6% of the visits. Similarly, in most of the visits, the MC rated the care environment of the health facility as friendly (79.2%) and within and outside the facility as very clean (60.4% and 56.3%, respectively). Only 4.9% of the MC visits had separate waiting rooms for adolescents, and posters and other health communication materials on STI and SRH were observed in only 39.6% of the visits.

**Table 1.** Summary statistics of the quality of care items.

Quality of care items	Yes	No
**Accessibility**		
Did you find the facility easily?	99 (68.8)	45 (31.3)
Were there any directional signs outside the facility?	96 (66.7)	48 (33.3)
Were there any directional signs within the facility?	64 (44.4)	80 (55.6)
**Acceptability of services**		
Was the outside of the facility clean? *(Very clean/Somewhat clean)*	87 (60.4)	57 (39.6)
Was the inside of the facility clean? *(Very clean/Somewhat clean)*	81 (56.3)	63 (43.8)
Was there a separate waiting room for adolescents?	7 (4.9)	137 (95.1)
Were there posters on STDs and other SRH issues in the facility?	57 (39.6)	87 (60.4)
How did you feel about the waiting time? *(Just OK/Too long)*	127 (88.2)	17 (11.8)
**Competencies and motivation of health workers**		
Was your medical history taken?	14 (9.7)	130 (90.3)
Was your social record taken?	52 (36.1)	92 (63.9)
Was your sexual/reproductive history taken?	67 (46.5)	77 (53.5)
Were you asked about previous contraceptive use experience?	49 (34)	95 (66)
**Privacy and confidentiality**		
Were you assured of confidentiality?	77 (53.5)	67 (46.5)
Were you counseled in a place where visual privacy was guaranteed?	53 (36.8)	91 (63.2)
Were you counseled in a place where auditory privacy was guaranteed?	78 (54.2)	66 (45.8)
Were you counseled on pregnancy prevention?	82 (56.9)	62 (43.1)
Were you counseled on any contraceptive methods?	96 (66.7)	48 (33.3)
Did the provider talk about HIV/AIDS with you?	44 (30.6)	100 (69.4)
Did you feel the provider had adequate time for you during consultation?	106 (73.6)	38 (26.4)
Did the provider give you an opportunity to ask questions?	114 (79.2)	30 (20.8)
**Global assessment**		
What did you think about the cost? *(No cost or affordable/Expensive)*	143 (99.3)	1 (0.7)
In general, how did you find the counseling? *(Satisfactory/Not Satisfactory)*	98 (68.1)	46 (31.9)
Will you recommend this facility to any of your colleague youth?	99 (68.8)	45 (31.3)
Did the HW appear knowledgeable about the case you presented	123 (85.4)	21 (14.6)
Did he/she address your worries seriously	101 (70.1)	43 (29.9)
Did he/she explain to you adequately	101 (70.1)	43 (29.9)
Time spent *(30 min or less/>30 min)*	119 (82.6)	25 (17.4)

The MC’s medical history, social record, sexual/reproductive history, or contraceptive experience was not obtained in most visits (90.3%, 63.9%, 53.5%, and 66.0%, respectively). In most visits, the MC felt confidentiality (53.5%) and auditory privacy (54.2%) were not provided. There was counseling on pregnancy prevention (56.9%), specifically on condoms (76.4%) but less frequently on sexual abstinence (47.2%) or HIV/AIDS (30.6%). In most visits also, the MCs felt the provider had adequate time for them (73.6%) and provided an opportunity to ask questions (79.2%), but very few were given educational materials (4.9%). Globally, most of the MC assessments on counseling were favourable (68.1%), and the MC would recommend the facility to a colleague in 68.8% of the visits. No cost was incurred in the majority of the visits (90.3%), and 82.6% spent a total of 30 minutes or less in the facility from arrival to departure.

### Factors associated with quality of care

Compared to the scenario about requesting information about STIs, the scenario about requesting counseling regarding premarital intercourse had a higher AHQOC index on average and was statistically significant both in simple and in the multiple linear models (β = 2.3 (0.1–4.6), p = 0.039 & β = 3.0(1.0–5.0), p = 0.003, respectfully) (Table 2). Also, an increase in the age of MC by one year was associated with a lower AHQOC score by 0.07 unit in the multiple linear model, but this was not statistically significant.

**Table 2.** Association between the AHQOC score and selected characteristics of the mystery clients, healthcare providers, and health facilities.

Selected characteristics	Simple linear coefficients‡		Multiple linear coefficients	
	β (95% CI)	p	β (95% CI)	p
**Scenario enacted**				
Requesting Information about STIs*				
Condom request	−0.7 (−2.7–1.2)	0.450	0.5 (−1.0–2.0)	0.510
Information regarding contraceptives	0.8 (−1.2–2.7)	0.445	0.2 (−1.3–1.7)	0.817
Counseling regarding premarital intercourse	2.3 (0.1–4.6)	0.039	3.0 (1.0–5.0)	0.003
**MC age**	0.0 (−0.5–0.6)	0.898	−0.1 (−0.7–0.5)	0.813
**MC sex**				
Male*				
Female	−0.3 (−1.6–1.0)	0.687	-	-
**Health worker sex**				
Male*				
Female	−0.5 (−2.9–1.9)	0.676	-	-
**MC sex # Health worker sex**				
Male MC#Male HW*				
Male MC#Female HW	−0.4 (−3.1–2.2)	0.748	−0.5 (−2.3–1.2)	0.554
Female MC#Male HW	−0.5 (−5.5–4.5)	0.847	0.5 (−2.9–3.9)	0.779
Female MC#Female HW	−0.6 (−3.2–1.9)	0.632	−1.1 (−3.0–0.8)	0.269
**Locality of facility**				
Urban*				
Rural	−2.7 (−5.1–0.2)	0.031	−0.4 (−1.8–1.0)	0.566
**Level of facility**				
Primary health center*				
Secondary Health facility	2.1 (−1.5–5.8)	0.247	0.8 (−1.3–2.8)	0.478
**MC ranking of health worker^**	1.9 (1.6–2.1)	<0.001	1.8 (1.5–2.1)	<0.001
**Constant**			14.4 (2.7–26.1)	0.016

^*^Reference categories; # Interaction terms; ^*^*^ *Ranking of proficiency of health worker encountered by MC on the scale of 1–10;* ^***‡***^*Constant term not reported*

For every unit increase in the ranking of the health working between 1 and 10, the AHQOC index increased by 1.8 units and this finding was statistically significant (p < 0.001). Female MC visits had a lower AHQOC rating on average than males by 0.3 units, and visits in which a female health worker was encountered had a lower AHQOC rating on average than male health workers by 0.5 units. However, both findings were not statistically significant. An interaction term between the sex of MC and health workers in the multiple linear model showed that female MC visits with female health workers had the lowest AHQOC rating, followed by male MC visits with female health workers. These were not statistically significant as well. The secondary health facilities were associated with higher AHQOC though these findings were also not statistically significant. The more rural LGA facilities had lower AHQOC on average but these findings were not statistically significant.

### Findings from the qualitative summaries

**Figure 1.**
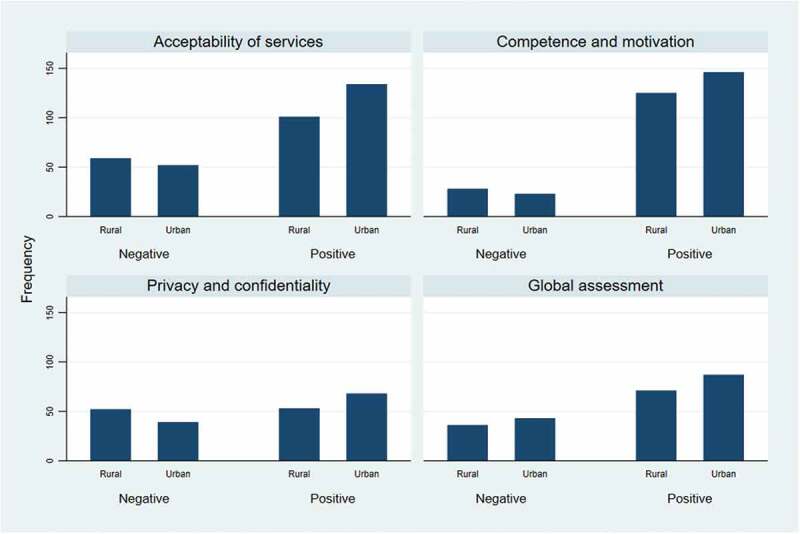
Bar chart showing sentiments analysis of responses the mystery clients to open-ended questions on care experiences during their clinic encounters.

From 144 transcripts, 1262 coded subsections were obtained, with 902 having positive sentiments and 360 having negative sentiments. Comparatively, MCs in the urban LGA had a higher frequency of occurrence of positive sentiments for responses to the question on the acceptability of services, competence and motivation of staff, privacy and confidentiality, and global assessment of the services provided (Figure 1). Also, MCs in the urban LGA had a lower frequency of occurrence of negative sentiments for acceptability of services, competence and motivation of staff, and privacy and confidentiality.

Some of the negative sentiments expressed concerning the acceptability of services included poor access roads to the facility, the facility being difficult to locate, unkempt external and internal environments, and generally unsatisfactory attitudes of the health workers. Positive sentiments included the health facility settings being appealing/welcoming/acceptable, prompt attention from health workers, the internal and external environment being neat, having directional signposts outside, or within the facility, having educative posters on walls of the facility, and the easy location of facility. Concerning competence and motivation of health workers, negative sentiments stated included health worker not providing counselling or health education, not being friendly or not knowledgeable, lack of competence concerning the presenting complaints of the MC. Positive sentiments expressed included highly motivated, knowledgeable and friendly health workers, and some maintenance of privacy and confidentiality during consultations. For global assessment of the MC visits, negative sentiments expressed by the MC included the facility not being youth friendly, lacking in personnel, and the services generally not being satisfactory. On the positive side, some of the MCs felt some facilities were youth-friendly and very accommodating, and they would recommend the services to their peers. Table 3 shows a selection of positive and negative sentiments expressed by the MC. Some of the characteristics that made the facility or provider youth-friendly from the perspective of the MC included physical accessibility of the facility, directional signpost/signage inside and outside the facilities, cleanliness of the internal and external environment of the facilities, placement of educative posters/materials on the wall of the facilities, friendliness of the health workers, knowledgeability of the health workers about the case presented, and provisions for privacy and confidentiality.

**Table 3.** Positive and negative sentiments for qualitative narratives of summary of MC visits.

Thematic Area	Negative sentiment	Positive Sentiment
**Acceptability of services**	*‘The environment was not quite appealing. There are lots of bush around the area. It was not easily located. The procedure was not acceptable to me. The settings was quite not appealing to patient.’*	*‘There were directional signs from the entrance of the building to the health post as it shares same building with other organizations. There were posters on health issues and awareness inside the facility for educational purposes. The departments were well indicated with a descriptions.’*
**Competence and motivation**	*‘The health provider wasn’t motivated to provide health service to youths at all and doesn’t seem very knowledgeable about the case I presented to her … she never gave me a chance to get to know because she didn’t health educate or address my worries seriously, she was just busy hurling insults on youths, expressing her dissatisfaction about how we have become this present world. “You are having rubbish up and down with multiple partners and you are scared of infection, you better get serious with your life, settle down with one, and stop disturbing yourself” she said to me.’*	*‘The service provider was skilful and motivated as she was knowledgeable about the case I presented and also cleared my worries she was also friendly throughout our consultation … ’*
**Privacy and confidentiality**	*‘Privacy and confidentiality was poorly observed as I was attended to by 3 staffs at the same time and a trader who came to sell her commodities met us and the discussion still continued.’*	*‘I was provided to privacy in which no one was there only me and the health worker. There is confidentiality also which he assured me’*
**Global assessment**	*‘The facility, to me, is a complete mess. In fact, it’s nothing to write home about. The health workers and even the health provider there doesn’t seem to feel so much concerned about the health of their client, and I guess this may contribute to the reasons the people around the community weren’t even familiar with the facility. They weren’t friendly, non-attentive and lacks orientation and motivation concerning the health of young people in adolescent. They all need to be reoriented and set straight to be in order.’*	*‘My global assessment concerning the friendliness of the health facility to adolescent and young people is that, they are wonderful and accommodating.’*

## Discussion

This study presents findings from 144 MC visits to 27 primary and secondary facilities by 12 MCs in two LGAs in Ogun State, Nigeria. The MCs had favourable opinions for most of the quality-of-care assessment items; however, some issues stood out. Nearly all the visits did not have MCs experiencing separate waiting rooms for adolescents or finding posters stating the client’s rights. Also, there was low use of educational materials. Concerning adolescents’ waiting rooms, the argument has been made that it is more desirable to provide integrated rather than standalone services for ASRH [26]. Also, within the integrated services, there should be minimal physical separation from other routine services so that the AYP is not discouraged from attendance due to the ease of being identified as using SRH services. Having separate waiting rooms or youth corners is part of the requirement of the minimum service package for adolescent and youth-friendly services (AYFHS) in Nigeria [20]. The practicality of this requirement is in doubt since it will involve additional resources in a setting where there is already a shortage of essential services. The non-use of posters and other educational materials that addressed health issues for AYP in the health facilities is perhaps easier to improve. A lot of the posters in public health facilities are provided by donors and partners. The government can look into the deliberate effort to produce a wide range of health education materials that can be continually available in health facilities.

The MCs’ medical history, social record, sexual/reproductive history, or contraceptive experience were not obtained in most of the visits. Also, there was less focus on HIV/AIDS counseling. Without obtaining the basic background information from their clients, it will not be possible for health workers to provide optimum care, especially counseling. Geary et al [27] similarly reported in their MC study in South Africa that sexual health histories were not obtained, and counseling on HIV/STI prevention was rarely provided. Furthermore, the arrangement of the facility did not always facilitate visual privacy. The incompetence demonstrated by the health worker in this manner may be because of not enough regard for the presenting complaints of the MCs. Perhaps, for instance, the health workers did not find it necessary for full clinical engagement for an AYP who was merely requesting condoms. This gap in the clinical practices of the health workers may be an important area for intervention in which health workers are taught how to conduct a proper clinical interview of AYP. One such approach is the *H*ome, *E*ducation/Employment, *E*ating, *A*ctivities, *D*rugs, *S*exuality, *S*uicidal ideation, and *S*afety (HEEADSSS), a psychological interview for adolescents [28]. Such can be included in the in-service training programmes for health workers. It will be an approach where health workers are taught standard questions to ask all AYP, especially if they are in the health facility for matters relating to SRH. Understandably, going through the HEEADSSS framework for each AYP client will add substantial time to clinical encounters. However, given that clinical and diagnostic endeavours are investigative, the health worker must have a foundational framework, such as the HEEADSSS, by which they operate. The application of the framework can then be modified on a case-by-case basis. For instance, an AYP client that came to request condoms may not need a full interrogation, but once the health worker suspects that there might be other unresolved issues concerning which the client is not so forthcoming, then a switch to a full application of the framework becomes necessary. Otherwise, that visit will be a missed opportunity to intervene. In addition, it was good to see that most MC visits in this study did not incur a cost and that most visits lasted only 30 minutes or less. Efforts must continue to be made to ensure the absence of such barriers.

As in other studies [29,30], we found that younger AYP experienced the poorer quality of care in this research. Also, the female MC visits had a lower AHQOC index rating on average compared to males. The interaction term introduced further indicated a poorer quality of care for females MC by female health workers, but these findings were not statistically significant in this study. Geary et al [27] reported that male simulated clients generally reported more positive experiences than female simulated clients, and judgmental attitudes were more commonly exhibited towards females than male simulated clients. The systematic review by Chandra-Mouli et al [9] also reported that some MC studies found provider behaviour and attitudes dependent on sex with some showing that male MCs were more likely to report services as satisfactory compared to female MCs. These findings may be connected to what may be a maternal instinct in the female health workers to address young female clients as their daughters or sisters. As such, they provide clinical advice that applies the prevailing cultural norm of how young girls are supposed to behave in the community to their female clients rather than as health experts. In some of the off-record comments of MCs during debriefing, some health workers were said to preface their counseling with statements such as: *‘See, let me advise like you were my daughter … ’*. However, we could not include the age of the health workers in our study, which may have further confirmed these ‘maternalistic’ tendencies (i.e. the health worker being old enough to be the biological parent of the client). A South African study reported that consultation with older health workers was associated with more positive experiences [27]. Indeed, there are likely to be differences across different settings due to differences in cultural and societal norms around age and sex relations. The differential treatment of boys and girls may also be a product of strongly internalised gender inequalities that typically place more stringent moral requirements and sexual behaviour norms on young women than on young men [31,32]. The age and sex relations between health workers and AYP is an area for future inquiry.

From the regression analysis, the MC visits that presented the case of ‘counseling regarding premarital intercourse’ had the highest AHQOC index on average. We did not investigate further why this might be so, but it may be that it attracted the least negative attitude from the health worker. Further research can explore whether the different scenarios indeed attract different levels of negativity from service providers. Also, in this study, secondary health facility visits had a higher quality of care index than PHCs, while urban health facilities had a higher quality of care index than the rural ones. These findings were not statistically significant, but future studies can explore these associations, perhaps with larger sample size. Finally, a higher ranking of the health worker on a scale of 1–10 corresponds to a higher AHQOC index of the MC visit, and this finding was statistically very significant. Such a simple ranking might be a quick and valid metric for assessing the quality of care in ASRH research.

From the MC summary narratives, there were overwhelmingly more positive than negative sentiments. This may be an indication that some progress is being made in providing optimum quality SRH services for AYP in the study location. Many of the negative sentiments expressed were about the poor infrastructure of the health facilities visited. Attention therefore should be given to aesthetics. It is not enough to merely build health facilities; they should also be beautiful within and without to make them attractive to AYP. Whether they are standalone youth facilities or for the general population, aesthetics plays a large role in visitors’ experience of health treatment.

## Limitations of the study

This study had some limitations. It is impossible to simulate a complete clinic experience both for practical and ethical reasons; hence there was a limit to the scenarios that could be presented. Mystery client methodology is only feasible where physiological symptoms are not required to be present [33], and the scenarios we used in this study excluded such. Boyce and Neale [21] recommend that information on the provision of physical examinations or procedures should be collected through exit interviews with actual patients. Chandra-Mouli [9] concludes that despite this limitation in the MC methodology, it is still an essential source of information about facility performance and provider behaviour.

The information provided by the MC is limited by the ability of the MC to recall the clinic encounter. We tried to minimise this in this study by ensuring the MC exit interview was conducted as soon as possible after the encounter and certainly before proceeding to another health facility. Furthermore, the age presented in the scenarios was not always the actual age of the MC since this was a simulation. Also, being students in nursing school, the MC might have been more confident in their interaction with health workers than the typical AYP. In this case, we traded off between having a competent MC and demonstrating as much as possible all the features of a typical AYP. Generally, it is worth noting that MCs are probably more likely to be bolder in their encounters than the typical AYP since they were selected and trained, and know they are only merely acting.

Maintaining service providers’ non-awareness of being observed is critical to the MC approach, but literature has queried whether this constitutes a breach of privacy [34]. To the extent that: (1) personal information of the health workers interacted with were not collected, hence none of them can be identified; (2) encounters with MCs were not events different from what the health workers would ordinarily experience on a normal work day; (3) the health worker had autonomy over their activities; and (4) there no intentional maleficence committed on the part of the MC, we conclude that ethical principles were adhered to. On the matter of informed consent, none was obtained directly from the health workers encountered by the MCs, but the Medical Officer of Health as well as heads of the public health facilities in the study LGA were given prior notice of the research. Indeed, this is not the same thing as directly obtaining informed consent but in the special case of mystery client research, this may suffice as long as other ethical principles are upheld. To further engage the health workers, findings from this research will be disseminated within the study location as a means of feedback. Finally, because we covered only a portion of health facilities in two out of 20 LGAs in Ogun State, there is a limitation to the generalisability of our results, and they should be interpreted with caution.

## Conclusions

This study has highlighted some gaps in the provision of SRH services to AYP in public health facilities, including the competencies of the health workers and the use of educational materials in clinic encounters with young people. This study partly confirms findings from other studies that female health workers react differently than male health workers to AYP, and that female AYP are treated differently than male AYP concerning SRH. We also found that the assessment of the health worker corresponds to the quality-of-care assessment of the facility. Finally, the AHQOC index used may be a useful metric for benchmarking the quality of care overtime in the provision of sexual and reproductive health services for adolescents and young people.

## Paper context

We assessed the provision and experiences of care in sexual and reproductive health (SRH) services for adolescents and young people (AYP) in public health facilities using mystery clients. Our findings indicate that females and younger AYP experienced lower quality of care than males or older AYP, and the quality of care was lower in rural compare to urban health facilities. The index we used may be a useful benchmarking tool for assessing quality over time.
